# What Makes Eye Contact Special? Neural Substrates of On-Line Mutual Eye-Gaze: A Hyperscanning fMRI Study

**DOI:** 10.1523/ENEURO.0284-18.2019

**Published:** 2019-02-28

**Authors:** Takahiko Koike, Motofumi Sumiya, Eri Nakagawa, Shuntaro Okazaki, Norihiro Sadato

**Affiliations:** 1 Division of Cerebral Integration, Department of System Neuroscience, National Institute for Physiological Sciences (NIPS), Aichi 444-8585, Japan; 2 Department of Physiological Sciences, School of Life Sciences, The Graduate University for Advanced Studies (SOKENDAI), Hayama 240-0193, Japan; 3 Biomedical Imaging Research Center (BIRC), University of Fukui, Fukui 910-1193, Japan

**Keywords:** automatic mimicry, eye contact, fMRI, mirror neurons, shared attention

## Abstract

Automatic mimicry is a critical element of social interaction. A salient type of automatic mimicry is eye contact characterized by sharing of affective and mental states among individuals. We conducted a hyperscanning functional magnetic resonance imaging study involving on-line (LIVE) and delayed off-line (REPLAY) conditions to test our hypothesis that recurrent interaction through eye contact activates the limbic mirror system, including the anterior cingulate cortex (ACC) and anterior insular cortex (AIC), both of which are critical for self-awareness. Sixteen pairs of human adults participated in the experiment. Given that an eye-blink represents an individual’s attentional window toward the partner, we analyzed pairwise time-series data for eye-blinks. We used multivariate autoregression analysis to calculate the noise contribution ratio (NCR) as an index of how a participant’s directional attention was influenced by that of their partner. NCR was greater in the LIVE than in the REPLAY condition, indicating mutual perceptual–motor interaction during real-time eye contact. Relative to the REPLAY condition, the LIVE condition was associated with greater activation in the left cerebellar hemisphere, vermis, and ACC, accompanied by enhanced functional connectivity between ACC and right AIC. Given the roles of the cerebellum in sensorimotor prediction and ACC in movement initiation, ACC–cerebellar activation may represent their involvement in modulating visual input related to the partner’s movement, which may, in turn, involve the limbic mirror system. Our findings indicate that mutual interaction during eye contact is mediated by the cerebellum and limbic mirror system.

## Visual Abstract

**Figure:**
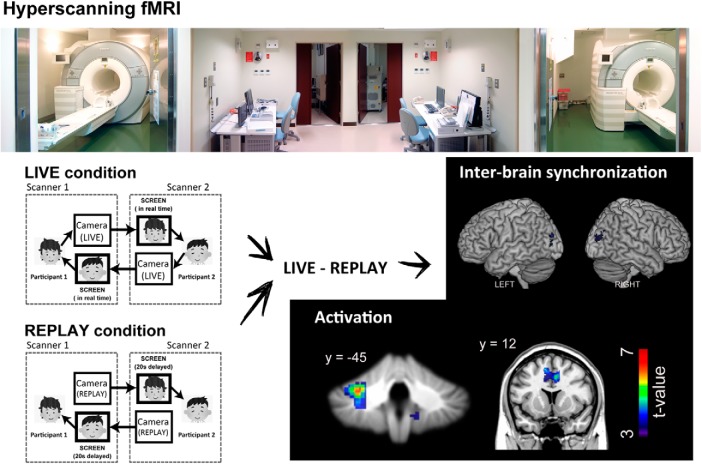

## Significance Statement

Eye contact is a key element that connects humans during social communication. We focused on a previously unaddressed characteristic of eye contact: real-time mutual interaction as a form of automatic mimicry. Our results indicate that real-time interaction during eye contact is mediated by the cerebellum and limbic mirror system. These findings underscore the importance of the mirror system and cerebellum in real-time unconscious social interaction.

## Introduction

Automatic mimicry refers to unconscious or automatic imitation of movement (Prochazkova and Kret, 2017). It is a critical part of human social interaction because it is closely tied to the formation of relationships and feeling of empathy (Chartrand and van Baaren, 2009). Automatic mimicry occurs when two or more individuals engage in the same behavior within a short window of time (e.g., facial expressions, body postures, laughter, yawning; Prochazkova and Kret, 2017). Automatic mimicry induces synchronous behavior through recurrent interaction (Okazaki et al., 2015), thereby enabling spontaneous synchronization (e.g., clapping) and goal-directed cooperation (Sebanz et al., 2006).

Eye contact is one of the most salient types of automatic mimicry, as two people must be able to synchronize their eye movements to make eye contact (Prochazkova and Kret, 2017). Eye gaze provides a communicative signal that transfers information regarding emotional and mental states (Emery, 2000). Eye contact, or mutual gaze, conveys the message, “I am attending to you,” thereby promoting effective communication and enhancing social interaction (Farroni et al., 2002; Schilbach, 2015).

Recent functional magnetic resonance imaging (fMRI) studies have revealed that eye contact activates the social brain, including the fusiform gyrus (George et al., 2001; Calder et al., 2002; Pageler et al., 2003), anterior superior temporal gyri (Calder et al., 2002; Wicker et al., 2003), posterior superior temporal gyri (Pelphrey et al., 2004; Schilbach et al., 2006; Conty et al., 2007), medial prefrontal cortex (Calder et al., 2002; Kampe et al., 2003; Schilbach et al., 2006; Conty et al., 2007), orbitofrontal cortex (Wicker et al., 2003; Conty et al., 2007), and amygdala (Kawashima et al., 1999; Wicker et al., 2003; Sato et al., 2004; for review, see Senju and Johnson, 2009). The above-mentioned studies were conducted using single-participant fMRI data, contrasting the neural activation elicited by an eye-contact event with that elicited by an eye-aversion event. However, neural substrates underlying recurrent interaction during eye contact that result in the development of shared, pair-specific psychological states (e.g., attention and emotion) remain unknown.

The mirror neuron system plays a role during mutual interaction through joint attention (Saito et al., 2010; Koike et al., 2016). The existence of two main networks with mirror properties has been demonstrated, with one residing in the parietal lobe and premotor cortex plus caudal part of the inferior frontal gyrus (parietofrontal mirror system), and the other formed by the insula and anterior medial frontal cortex (limbic mirror system; Cattaneo and Rizzolatti, 2009). The parietofrontal mirror system is involved in recognizing voluntary behavior, while the limbic mirror system is devoted to recognizing affective behavior (Cattaneo and Rizzolatti, 2009). We hypothesized that mutual interaction involving eye contact activates the limbic mirror system.

This study aimed to elucidate the behavioral and neural representations of mutual interaction during eye contact using hyperscanning fMRI (Koike et al., 2016). The neural activity associated with real-time eye contact was compared with that of non-real-time eye contact using a double-video system (Murray and Trevarthen, 1985). Eye contact is characterized by a two-way, behavioral stimulus-to-brain coupling, such that the behavior of a partner is coupled to the activation in the brain of the other (Hari and Kujala, 2009). Thus, face-to-face interaction through eye contact can be regarded as a mirrored reactive–predictive controller system consisting of two controllers (Wolpert et al., 2003). We used eye-blink as a behavioral index of mutual exchange of communicative cues between two participants during eye contact. As the blinks of others can be easily recognized due to their relatively long duration (200–400 ms; VanderWerf et al., 2003), eye-blinks can provide social communication cues (Nakano and Kitazawa, 2010). Further, blink rates change with internal states such as arousal, emotion, and cognitive load (Ponder and Kennedy, 1927; Hall, 1945; Stern et al., 1984). Finally, the timing of eye-blinks is associated with implicit (Herrmann, 2010) and explicit (Orchard and Stern, 1991) attentional pauses in task content. Nakano and Kitazawa (2010) observed that eye-blinks of a listener and speaker were synchronized during face-to-face conversations, and concluded that eye-blinks define the attentional temporal window and that its synchronization reflects smooth communication between interactants through sharing of attention in the temporal domain. In this study, we used hyperscanning fMRI to analyze brain activation related to eye-blinks using the following different measures: activation, modulation of functional connectivity, and interbrain synchronization.

## Materials and Methods

### Participants

Thirty-four volunteers participated in the experiment (20 men, 14 women; mean age ± SD, 21.8 ± 2.12 years). Participant pairs were determined before the experiment and consisted of participants of the same sex. None of the participants had met each other before the experiment. All participants except one were right handed, as evidenced by the Edinburgh Handedness Inventory (Oldfield, 1971). None of the participants had a history of neurologic or psychiatric illness. The protocol was approved by the ethics committee of the National Institute for Physiological Sciences. The study was conducted in compliance with the national legislation and the Code of Ethical Principles for Medical Research Involving Human Subjects of the World Medical Association (Declaration of Helsinki). All participants provided written informed consent before the experiment.

### Design and Procedure

#### Experimental setup

To measure neural activation during the on-line exchange of eye signals between pairs of participants, we used a hyperscanning paradigm with two MRI scanners (Magnetom Verio 3T, Siemens) installed side-by-side in parallel, sharing one control room and a triggering system (Morita et al., 2014; Koike et al., 2016). The top component of the standard 32-channel coil was replaced by a small four-channel flex coil (Siemens) attached with a special holding fixture (Takashima Seisakusho; Morita et al., 2014; Koike et al., 2016) to fully visualize the eye region. On-line grayscale video cameras were used during scanning to identify reciprocal face-to-face interaction (NAC Image Technology). The cameras captured images of each participant’s face, including the eyes and eyebrows. The captured images were in turn projected using a liquid crystal display projector (CP-SX12000J, Hitachi) onto a half-transparent screen that stood behind the scanner bed. The captured images were also entered into the picture delay system (VM-800, Sugioka System), which could output video delayed by an arbitrary amount of time. For analysis, video pictures used in the experiment were transferred to a video recording system (Panasonic). We recorded facial movement in AVI (audio video interleave) format (640 × 480 pixels, 30 frames/s). While the exact values varied depending on the participant’s head size, the screen stood ∼190 cm from the participants’ eyes, and the stimuli were presented at a visual angle of 13.06° × 10.45°. The delay between the capture and projection of the participants’ face was controlled using a hardware device (VM-800, Ito Co., Ltd.) connected between the video camera and projector. The delay was set at 20 s for the REPLAY condition and 0 s for the LIVE condition. The intrinsic delay of the on-line video system in this experimental setup was ∼100 ms.

#### Experimental conditions

**Figure 1.**
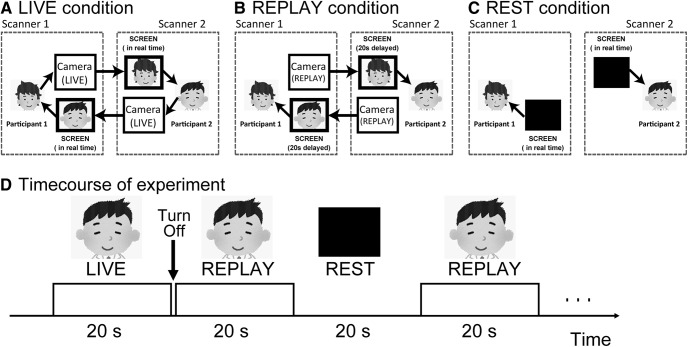
Experimental setup. ***A***, LIVE condition: the face of Participant 1 is projected on the screen of Participant 2 in real time and vice versa, allowing a mutual exchange of information. ***B***, REPLAY condition: the picture is projected on the screen with a 20 s delay; therefore, there is no mutual interaction between participants in real time. ***C***, REST condition (baseline): no image is presented on the black screen. ***D***, Sequence of presentation of the experimental conditions.

We adopted a conventional blocked design for this study. Each run included three conditions: LIVE, REPLAY, and REST. During the LIVE condition, participants were presented with a live video of their partner’s face in real time (Fig. 1*B*), allowing for the on-line exchange of information between the two participants. We instructed participants to gaze into the right or left eye of their partners and think about their partner as follows: what he/she is thinking about, what is his/her personality, how he/she is feeling. The participants were instructed not to exhibit explicit facial expressions such as laughing or grimacing. We also informed them that we will stop MRI scanning if they were not gazing into the partner’s eyes for an extended period of time. The REPLAY condition was identical to the LIVE condition, except that the participant watched a video picture of their partner’s face presented at a delay of 20 s. Therefore, there was no real-time interaction between the participants (Fig. 1*C*). During the REPLAY condition, the participant was informed that all the videos they were watching represented their partner’s face in real time. During the REST condition (baseline), participants were required to gaze at the blank screen (Fig. 1*A*). Although we monitored the participants to ensure that they do not fall asleep, two participants fell asleep during the experiment, and we had to restart the experiment after a short break.

Before starting the run, a live video of the partner was presented on the screen to confirm that an interactive partner was in the other scanner. Following confirmation, the video was turned off. The first run began with the REST condition for 30 s, followed by the LIVE, REPLAY, and REST conditions for 20 s each. After each 20 s presentation of the partner’s face, the screen was turned off for 1 s, and the condition was switched (e.g., from LIVE to REPLAY, REPLAY to REST; Fig. 1*D*). The 1 s interval was designed to prevent participants from becoming aware of the difference between the LIVE and REPLAY conditions. The order of presenting the conditions was pseudorandomized. The conditions were switched manually during the fMRI run according to a predefined experimental design. Each run consisted of eight LIVE and eight REPLAY conditions. The total length of each run was 8 min and 30 s, and the entire scan consisted of four runs. Throughout the experiment, none of the participants exhibited any sudden display of emotions such as laughter.

An interview following the experiment revealed that only one female pair realized that a delayed facial picture was presented in one of the conditions during the experiment; thus, the requirements of the experiment were not fulfilled in the pair. Data were analyzed from the remaining 32 participants (20 men, 12 women; mean ± SD age, 21.8 ± 2.03 years).

#### MRI data acquisition

Brain activation data were acquired using interleaved T2*-weighted, gradient echo, echoplanar imaging (EPI) sequences. Volumes consisted of 60 axial slices, each 2.0 mm thick with a 0.5 mm gap, covering the entire cerebral cortex and cerebellum. The time interval between two successive acquisitions of the same image [repetition time (TR)] was 1000 ms, with a flip angle of 80° and echo time (TE) of 30 ms. The field of view (FOV) was 192 mm, and the in-plane matrix size was 64 × 64 pixels. We used the multiband accelerated sequence developed at the University of Minnesota (Moeller et al., 2010), with the multiband factor set to 6. Thus, 510 volumes (8 min and 30 s) were collected for each run. For anatomic reference, T1-weighted high-resolution images were obtained using a three-dimensional magnetization-prepared rapid acquisition gradient echo (MPRAGE) sequence (TR = 1800 ms; TE = 2.97 ms; FA = 9°; FOV = 256 mm; voxel dimensions = 1 × 1 × 1 mm^3^) and a full 32-channel phased array coil.

### Data analysis

#### Behavioral data analysis

##### Extraction of eye-blink time series

Eye-blink was chosen as a behavioral index of interaction during mutual gaze (Koike et al., 2016). We calculated the “motion energy” using the AVI video of the participant’s face during the task (Schippers et al., 2010) to evaluate the time series of eye-blinks. Due to technical difficulties with the video recording system, data from two pairs were unavailable. In total, video data of faces from 14 pairs (18 men, 10 women; mean ± SD age, 21.8 ± 2.17 years) were subjected to the analysis described below.

**Figure 2.**
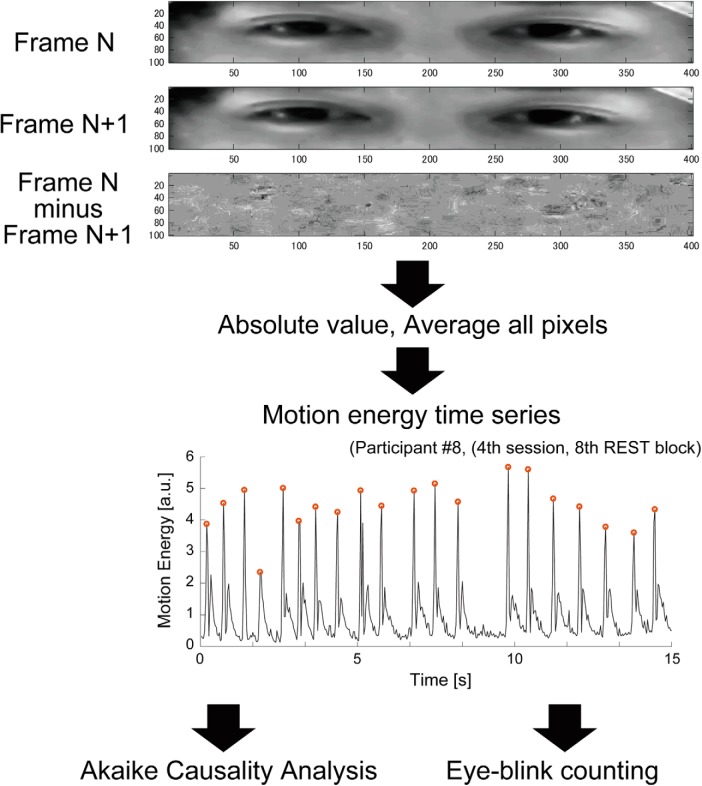
Evaluation of the motion energy time series representing eye-blinks. The red dots indicate the timing of the detected eye-blink.

Figure 2 illustrates the procedure used to calculate the motion energy time series representing eye-blinks. First, the spatial window (400 × 100 pixels) of the AVI video was manually set to cover the eye area of each participant. Second, using the pixel intensity of the defined eye area, we obtained the motion energy index, which can detect the occurrence of motion only from a series of pictures (Schippers et al., 2010). The first-order difference in picture intensity was calculated frame by frame in each pixel, and the average of the absolute value of differences in each frame was calculated. This process was used to obtain motion energy values at specific time points. The calculation was repeated to obtain the motion energy time series reflecting eye-blinks during each run. Third, we divided the time series in each run into shorter subsections corresponding to the LIVE, REPLAY, and REST conditions. Although each condition lasted 20 s (Fig. 1*D*), we analyzed only the final 15 s of each condition to minimize the effect of brightness instability (largely due to the procedure for switching conditions). We obtained eight time series for each condition of a single run. As each participant underwent four runs, 32 time series were obtained for each condition per participant. Finally, the effect of the linear trend in the data was removed using the “detrend” function implemented in MATLAB. The whole procedure was performed using a MATLAB script (MATLAB 14, MathWorks) developed in-house.

##### Number of eye-blinks

To determine whether the number of eye-blinks itself was influenced by differences in the type of task, we calculated the number of eye-blinks in the LIVE, REPLAY, and REST conditions using the extracted time series of motion energy. We first adapted the peak-detection function implemented in MATLAB, which automatically detected and marked the time point at which the eye-blink appeared to occur (Fig. 2). Next, we visually examined whether the detected time point was acceptable. Finally, we calculated the average number of eye-blinks in 1 block (15 s) for each participant. All calculations were performed using a MATLAB script (MATLAB 2014) developed in-house.

##### Causality analysis between eye-blink time series

Several hyperscanning studies have used synchronization or correlation as an index of interaction (Koike et al., 2016), neither of which can evaluate the directional effect. In this study, we used an Akaike causality model (Akaike, 1968; Ozaki, 2012), which can delineate the causal direction and quantify its effect. The Akaike causality model uses a multivariate autoregressive (MVAR) model under the steady-state assumption and can quantify the proportion of the power-spectral density of an observed variable from the independent noise of another variable. The quantified causality, that is, the noise contribution ratio (NCR) index, is regarded as a measure of how one variable is influenced by another. In this study, we assumed that the eye-blink time series satisfies a steady-state assumption at least in one block. The NCR values were calculated as follows.

First, an MVAR model was applied to a pair of time-series data, *x*(*t*) and *y*(*t*), using the linear sum of the history of the two time series, as follows:

(1) $$x\left(t\right)=\sum_{i=1}^{N}a_{i}x\left(t-i\right)+\sum_{i=1}^{N}b_{i}y\left(t-i\right)+u_{x}\left(t\right)$$

(2) $$y\left(t\right)=\sum_{i=1}^{N}c_{i}x\left(t-i\right)+\sum_{i=1}^{N}d_{i}y\left(t-i\right)+u_{y}\left(t\right),$$

where the time series x(t) and y(t) correspond to the time series of the participant’s eye-blinks and that of the partner, respectively. In these equations, $a_{i}$, $b_{i}$, $c_{i}$, and $d_{i}$ indicate AR coefficients, while $u_{x}$ and $u_{y}$ indicate the residual noise in the eye-blinks of the participant and partner, respectively. The AR order *N* defines the duration of the history. For each pair of time-series data, the AR order *N* was estimated to minimize the Akaike information criterion in the range from 1 to 10. Next, we estimated the power spectrum of the two time series based on the sum of the contributions of the *x*-specific noise (i.e., ${\left|\alpha \left(f\right)\right|}^{2}\sigma_{ux}{}^{2}$) and *y*-specific noise (i.e., ${\left|\beta \left(f\right)\right|}^{2}\sigma_{uy}{}^{2}$). Here, |α(f)| and |I2(f)| are frequency response functions, derived from Fourier transformation via an impulse response function, using a set of AR coefficients, while $\sigma_{ux}$ and $\sigma_{ux}$ indicate the variance of residual noise $u_{x}$ and $u_{y}$, respectively. The $NCR_{y\to x}\left(f\right)$, an index reflecting how the participant’s eye-blinks x(t) are influenced by the partner’s eye-blinks y(t), was calculated from the ratio of part of the spectral density of x(t) contributed by $\sigma_{uy}{}^{2}$ to the total spectral density of x(t) at frequency *f*. Therefore, $NCR_{y\to x}\left(f\right)$ can be expressed as follows:

(3) $$NCR_{y\to x}\left(f\right)=\frac{{\left|\beta \left(f\right)\right|}^{2}\sigma_{uy}{}^{2}}{{\left|\alpha \left(f\right)\right|}^{2}\sigma_{ux}{}^{2}+{\left|\beta \left(f\right)\right|}^{2}\sigma_{uy}{}^{2}}.$$

To assess how x(t) is influenced by y(t) across the whole frequency range, we mathematically integrated *NCR* values via trapezoidal numerical integration as follows:

(4) $$\Sigma NCR_{y\to x}=\int_{0}^{f_{s}/2}NCR_{y\to x}\left(f\right)df,$$

where *f_s_* is the sampling frequency of the time series x(t) and y(t). In this study, *f_s_* was 30 Hz, based on the frame rate of the video data. We collected 32 time series for each condition. Therefore, our calculations yielded 32 ΣNCR values for each condition per participant. These 32 ΣNCR values were averaged to calculate one summarized ΣNCR value for each participant in each condition. Using the summarized ΣNCR, we applied statistical analyses to determine whether the influence of the partner differed between conditions. The entire procedure was performed using a MATLAB script (MATLAB 2014) written in-house.

In this study, we calculated four ΣNCR values to assess how a participant’s eye-blink was influenced by that of the partner. Firstly, in the REST condition, participants could see nothing on the screen. Therefore, the ΣNCR value in the REST condition (i.e. ${\text{ΣNCR}}_{F\to F}^{REST}$) was regarded as a baseline of causal relationship. In the LIVE condition, the face of one participant was immediately projected on the screen, and the partner was able to see the face in real time. In this condition, we calculated ΣNCR between two participants’ time series (i.e., ${\text{ΣNCR}}_{F\to F}^{LIVE}$).　The ΣNCR value represents how participants influence their partners when they mutually interact with each other in real time. Next, in the REPLAY condition, two types of causality were calculated as follows: first, the ΣNCR value between actual eye-blinks, like in the LIVE condition (i.e., ${\text{ΣNCR}}_{F\to F}^{REPLAY}$); and second, the ΣNCR value in the REPLAY condition representing how the eye-blinks projected on the screen has an influence on the actual eye-blink time series, ${\text{ΣNCR}}_{S\to F}^{REPLAY}$. While it is possible that a participant’s face receives influence from the delayed picture on the screen (Nakano and Kitazawa, 2010), influence from an actual eye-blink to the screen (reverse influence) is theoretically absent. We also calculated the ΣNCR value (i.e., ${\text{ΣNCR}}_{F\to F}^{REST}$). It represents how participants are influenced by a video picture, while there could be only unidirectional influence from the screen to actual eye-blinks.

##### Estimation of statistical inferences and data visualization

All statistical inference estimation for the behavioral data analysis was performed using R (RRID:SCR_001905). We analyzed three types of behavioral measures. (1) The number of eye-blinks is highly influenced by the degree of attention (Ponder and Kennedy, 1927; Hall 1945; Stern et al., 1984; Orchard and Stern, 1991; Herrmann, 2010) and could reflect the differences across conditions. We tested the number of eye-blinks in three conditions using repeated-measures analysis of variance (ANOVA). (2) ΣNCR values: we have four ΣNCR values for each participant, ${\text{ΣNCR}}_{F\to F}^{REST}$ in the REST condition, ${\text{ΣNCR}}_{F\to F}^{REPLAY}$ and ${\text{ΣNCR}}_{S\to F}^{REPLAY}$ in the REPLAY condition, and ${\text{ΣNCR}}_{F\to F}^{LIVE}$ in the LIVE condition. The differences between them were assessed using repeated-measures ANOVA. (3) Enhanced ΣNCR values: in the REST condition, participants know there is no interaction with a partner as nothing is projected on the screen. Therefore, theoretically speaking, the REST condition could be regarded as a baseline condition. We calculated the increase in ΣNCR values (enhancement) by subtracting the ${\text{ΣNCR}}_{F\to F}^{REST}$ value from each of the ΣNCR values. Thus, we have three enhanced ΣNCR values for each participant: ${\text{ΣNCR}}_{F\to F}^{LIVE}-{\text{ΣNCR}}_{F\to F}^{REST},$ ${\text{ΣNCR}}_{F\to F}^{REPLAY}-{\text{ΣNCR}}_{F\to F}^{REST},$ and ${\text{ΣNCR}}_{S\to F}^{REPLAY}-{\text{ΣNCR}}_{F\to F}^{LIVE},$. Repeated-measures ANOVA was used to test the differences between these values. In all ANOVA procedures, the effect size was measured using the generalized η^2^ value (Olejnik and Algina, 2003). In the *post hoc* pairwise analysis, estimated *p* values were adjusted using a Bonferroni correction. The confidence levels for *post hoc* pairwise analyses were calculated via the pairwise confidence intervals of Franz and Loftus (2012). The details of the statistical methods used in this behavioral data analysis are listed in Table 1. All the graphs were prepared using the RainCloudPlots R-script (Allen et al., 2018; https://github.com/RainCloudPlots/RainCloudPlots), which could provide a combination of box, violin, and dataset plots. In the dataset plot, each dot represents a data point, respectively. Outliers were defined by 2 SDs and are represented in Figure 2 by red diamonds. In the boxplot, the line dividing the box represents the median of the data, while the ends of the box represent the upper and lower quartiles. The extreme lines show the highest and lowest values excluding outliers defined by 2.0 SDs.

**Table 1.** Statistical analysis

Manuscript	Figure	Data type	Data structure	Type of test	Multiple comparison correction	Program	Statistics	*p* values	Power/confidence interval
a	3*A*	Number of eye-blinks	Normal distribution	One-way repeated ANOVA		R	*F*_(2,54)_ = 13.1814	***p* < 0.0001**	η_g_^2^ = 0.03540
b		Number of eye-blinks	Normal distribution	*t* test (*post hoc* test, LIVE vs REST)	Bonferroni	R	*t*_(27)_ = 3.9464	***p* = 0.0015**	mean = −1.2757 (−1.9389 to −0.6124)
c		Number of eye-blinks	Normal distribution	*t* test (*post hoc* test, REPLAY vs REST)	Bonferroni	R	*t*_(27)_ = 3.8499	***p* = 0.0021**	mean = −0.7946 (−1.2182 to −0.3711)
d		Number of eye-blinks	Normal distribution	*t* test (*post hoc* test, LIVE vs REPLAY)	Bonferroni	R	*t*_(27)_ = 2.3522	*p* = 0.0786	mean = −0.4810 (−0.9006 to −0.0614)
e	3*B*	Absolute ΣNCR	Normal distribution	One-way repeated ANOVA		R	*F*_(3,81)_ = 3.9830	***p* = 0.0295**	η_g_^2^ = 0.03236
f		Absolute ΣNCR	Normal distribution	Paired *t* test (*post hoc* test, LIVEFF vs REPLAYFF)	Bonferroni	R	*t*_(27)_ = 3.406	***p* = 0.0126**	mean = 1.2294 (0.4888–1.9700)
g		Absolute ΣNCR	Normal distribution	Paired *t* test (*post hoc* test, LIVEFF vs RESTFF)	Bonferroni	R	*t*_(27)_ = 1.4598	*p* = 0.9354	mean = 0.8888 (−0.3604 to 2.1379)
h		Absolute ΣNCR	Normal distribution	Paired *t* test (*post hoc*test, LIVEFF vs REPLAYSF)	Bonferroni	R	*t*_(27)_ = 3.2934	***p* = 0.0168**	mean = 1.0455 (0.3941–1.6969)
i		Absolute ΣNCR	Normal distribution	Paired *t* test (*post hoc* test, REPLAYFF vs RESTFF	Bonferroni	R	*t*_(27)_ = 0.9065	*p* = 1.0000	mean = −0.3406 (−1.1116 to 0.4304)
j		Absolute ΣNCR	Normal distribution	Paired *t* test (*post hoc* test, REPLAYFF vs REPLAYSF	Bonferroni	R	*t*_(27)_ = 1.2083	*p* = 1.0000	mean = −0.1838 (−0.4960 to 0.1284)
k		Absolute ΣNCR	Normal distribution	Paired *t* test (*post hoc* test, RESTFF vs REPLAYSF	Bonferroni	R	*t*_(27)_ = 0.4349	*p* = 1.0000	mean = 0.1568 (−0.5829 to 0.8965)
l		Absolute ΣNCR	Normal distribution	One-way repeated ANOVA		R	*F*_(3,69)_ = 4.3334	***p* = 0.0074**	η_g_^2^ = 0.0785
m		Absolute ΣNCR	Normal distribution	Paired *t* test (*post hoc* test, LIVEFF vs REPLAYFF)	Bonferroni	R	*t*_(23)_ = 3.0965	***p* = 0.0306**	mean = 1.0291(0.3416–1.7165)
n		Absolute ΣNCR	Normal distribution	Paired *t* test (*post hoc* test, LIVEFF vs RESTFF)	Bonferroni	R	*t*_(23)_ = 1.0783	*p* = 1.0000	mean = 0.4588 (−0.4214 to 1.3390)
o		Absolute ΣNCR	Normal distribution	Paired *t* test (*post hoc* test, LIVEFF vs REPLAYSF)	Bonferroni	R	*t*_(23)_ = 3.0779	***p* = 0.0318**	mean = 0.7771(0.2548–1.2994)
p		Absolute ΣNCR	Normal distribution	Paired *t* test (*post hoc* test, REPLAYFF vs RESTFF	Bonferroni	R	*t*_(23)_ = 1.9902	*p* = 1.0000	mean = −0.5702 (−1.1630 to 0.0225)
q		Absolute ΣNCR	Normal distribution	Paired *t* test (*post hoc* test, , REPLAYFF vs REPLAYSF	Bonferroni	R	*t*_(23)_ = 1.4744	*p* = 0.9234	mean = −0.2519 (−0.6054 to 0.1015)
r		Absolute ΣNCR	Normal distribution	Paired *t* test (*post hoc* test, REPLAYFF vs REPLAYSF	Bonferroni	R	*t*_(23)_ = 1.1336	*p* = 1.0000	mean = 0.3183 (−0.2626 to 0.8992)
s	3*C*	Relative ΣNCR	Normal distribution	One-way repeated ANOVA		R	*F*_(2,54)_ = 10.3784	***p* = 0.0002**	η_g_^2^ = 0.0483
t		Relative ΣNCR	Normal distribution	Paired *t* test (*post hoc* test, LIVEFF vs REPLAYFF	Bonferroni	R	*t*_(27)_ = 3.4061	***p* = 0.0063**	mean = 1.2294 (0.4888–1.9700)
u		Relative ΣNCR	Normal distribution	Paired *t* test (*post hoc* test, LIVEFF vs REPLAYSF	Bonferroni	R	*t*_(27)_ = 3.2934	***p* = 0.0084**	mean = 1.0455 (0.3941–1.6969)
v		Relative ΣNCR	Normal distribution	Paired *t* test (*post hoc* test, REPLAYFF vs RESTSF	Bonferroni	R	*t*_(27)_ = 1.2083	*p* = 0.7122	mean = −0.1838 (−0.4960 to 0.1284)
w		Relative ΣNCR	Normal distribution	One-way repeated ANOVA		R	*F*_(2,40)_ = 7.9233	***p* = 0.0013**	η_g_^2^ = 0.1330
x		Relative ΣNCR	Normal distribution	Paired *t* test (*post hoc* test, LIVEFF vs REPLAYFF	Bonferroni	R	*t*_(20)_ = 2.8343	***p* = 0.0306**	mean = 7805(0.0102–0.0250)
y		Relative ΣNCR	Normal distribution	Paired *t* test (*post hoc* test, LIVEFF vs REPLAYSF	Bonferroni	R	*t*_(20)_ = 2.9034	***p* = 0.0264**	mean = 0.8362(0.0088–0.0167)
z		Relative ΣNCR	Normal distribution	Paired *t* test (*post hoc* test, REPLAYFF vs RESTSF	Bonferroni	R	*t*_(20)_ = 0.6790	*p* = 1.0000	mean = 0.0558 (−0.1156 to 0.2271)
aa		Absolute ΣNCR	Normal distribution	Repeated ANOVA, Main effect of conditions		R	*F*_(3,81)_ = 3.9830	***p* = 0.0106**	η_g_^2^ = 0.0132
bb		Absolute ΣNCR	Normal distribution	Repeated ANOVA, Main effect of sessions		R	*F*_(3,81)_ = 1.0351	*p* = 0.3816	η_g_^2^ = 0.0139
cc		Absolute ΣNCR	Normal distribution	Repeated ANOVA, Interaction (session x condition)		R	*F*_(9,243)_ = 1.8235	*p* = 0.0647	η_g_^2^ = 0.0128
dd	4	fMRI (BOLD activation)	Normal distribution	Paired *t* test (LIVE > REPLAY)	Random effect model at cluster-level inference	SPM			
ee		fMRI (BOLD activation)	No assumption	Paired *t* test (LIVE > REPLAY)	Nonparametric permutation test at cluster-level inference	SnPM			

ff	5	fMRI (PPI value)	Normal distribution	Paired *t* test (LIVE > REPLAY)	Random effect model at cluster-level inference	SPM			
gg		fMRI (PPI value)	No assumption	Paired *t* test (LIVE > REPLAY)	Nonparametric permutation test at cluster-level inference	SnPM			
hh	6	fMRI (normalized interbrain sync)	Normal distribution	Paired *t* test (LIVE > REPLAY)	Random effect model at cluster-level inference	SPM			
ii		fMRI (normalized interbrain sync)	No assumption	Paired *t* test (LIVE > REPLAY)	Nonparametric permutation test at cluster-level inference	SnPM			

#### Neuroimaging analysis

##### Image preprocessing

The first 10 volumes (10 s) of each fMRI run were discarded to allow for stabilization of the magnetization, and the remaining 500 volumes/run (total of 2000 volumes/participant) were used for the analysis. The data were analyzed using statistical parametric mapping (SPM12, Wellcome Trust Center for Neuroimaging, London, UK; RRID:SCR_007037) implemented in MATLAB 2014 (RRID:SCR_001622). All volumes were realigned for motion correction. The whole-head T1-weighted high-resolution MPRAGE volume was coregistered with the mean EPI volume. The T1-weighted image was normalized to the Montreal Neurologic Institute (MNI) template brain using a nonlinear basis function in SPM12. The same normalization parameters were applied to all EPI volumes. All normalized EPI images were spatially smoothed in three dimensions using a Gaussian kernel (full-width at half-maximum = 8 mm).

##### Estimation of task-related activation using univariate generalized linear modeling

Because of technical difficulties, we could not acquire fMRI data from one pair. Therefore, we analyzed whole fMRI data acquired from 30 participants (18 men, 12 women; mean ± SD age, 21.7 ± 2.10 years). Statistical analysis was conducted at two levels. First, individual task-related activation was evaluated. Second, summary data for each participant were incorporated into a second-level analysis using a random-effects model (Friston et al., 1999) to make inferences at a population level.

In the individual-level analysis, the blood oxygenation level-dependent (BOLD) time series representing the brain activation of each participant was first modeled using a boxcar function convolved with a hemodynamic response function and filtered using a high-pass filter (128 s), while controlling for the effect of runs. Serial autocorrelation assuming a first-order autoregressive model was estimated from the pooled active voxels using the restricted maximum likelihood procedure and used to whiten the data (Friston et al., 2002). No global scaling was applied. The model parameters were estimated using the least-squares algorithm on the high pass-filtered and whitened data and design matrix. Estimates for each of the model parameters were compared with the linear contrasts to test hypotheses regarding region-specific condition effects. Next, the weighted contrasts of the parameter estimate (i.e., LIVE > REST and REPLAY > REST) in the individual analyses were incorporated into the group analysis. Contrast images obtained via individual analyses represented the normalized task-related increment of the MR signal relative to the control condition (i.e., the REST condition) for each participant.

In the group-level analysis, we investigated differences in brain activation between the LIVE and REPLAY conditions using these contrast images and the random-effects model implemented in SPM12. We analyzed these data using the paired *t* test. The resulting set of voxel values for each contrast constituted a statistical parametric map of the *t* statistic (SPM {t}). **T**he threshold for significance of the SPM {t} was set at *p* < 0.05 with familywise error (FWE) correction at the cluster level for the entire brain (Friston et al., 1996). To control FWE rates using random field theory (Eklund et al., 2016), the height threshold was set at an uncorrected *p* value <0.001, which is conservative enough to depict cluster-level inference with the parametric procedure (Flandin and Friston, 2017). To validate the statistical inference with a parametric method, we also tested the statistical significance of activation using a nonparametric permutation test implemented in the SnPM13 toolbox (RRID:SCR_002092; Nichols and Holmes, 2002). We used the nonparametric paired *t* test with no variance smoothing; the number of permutations was set at 10,000. The SnPM toolbox did not yield statistical significance at all the voxels reported in SPM; thus, the *p* values for some voxels have not been listed in the tables.

##### Generalized psychophysiologic interaction analysis

**Figure 3.**
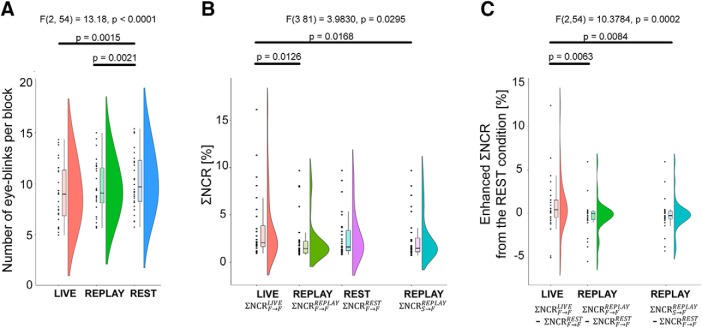
Behavioral analysis. ***A***, The number of eye-blinks per block. We omitted the first 5 s of each block because of instability of the recorded video induced by task switching; the number of eye-blinks was therefore calculated based on the succeeding 15 s. Each dot represents a data point. In the boxplot, the line dividing the box represents the median of the data, the ends represent the upper/lower quartiles, and the extreme lines represent the highest and lowest values excluding outliers. ***B***, ΣNCR values. The integral of the NCR of each condition across the whole frequency range was calculated. ${\text{ΣNCR}}_{F\to F}^{LIVE}$ is the ΣNCR from the time series of the participant’s facial movement to that of the partner during the LIVE condition. ${\text{ΣNCR}}_{F\to F}^{REPLAY}$ is the ΣNCR from the time series of the participant’s facial movement to that of the partner during the REPLAY condition. ${\text{ΣNCR}}_{F\to F}^{REST}$ is the ΣNCR from the time series of the participant’s facial movement to that of the partner during the REST condition. ${\text{ΣNCR}}_{S\to F}^{REPLAY}$ is the ΣNCR from the time series from the participant’s delayed facial movement on the screen to the partner’s time series during the REPLAY condition. ***C***, Enhanced ΣNCR values from the REST condition.

Next, we performed generalized psycho-physiologic interaction (gPPI) analysis (Friston et al., 1997; McLaren et al., 2012) using the CONN toolbox (Whitfield-Gabrieli and Nieto-Castanon, 2012; RRID:SCR_009550) to reveal how effective connectivity from the LIVE- or REPLAY-specific regions (toward other brain regions) was altered between the LIVE and REPLAY conditions. For this purpose, we selected three clusters based on the LIVE > REPLAY contrast defined by the results of univariate generalized linear modeling (GLM) analysis (Fig. 3, Table 2) as seed regions for the gPPI analysis. We used conventional seed-to-voxel gPPI analysis in which the whole brain is the search area. The components associated with a linear trend, CSF, white matter (WM), and experimental tasks (i.e., LIVE and REPLAY effects) were removed from the BOLD time series as confounding signals. Using the residual time series, gPPI analysis was performed to evaluate whether the effective connectivity from the seed region was modulated by the task condition (i.e., the LIVE or REPLAY condition) at the individual level. This individual-level analysis produced contrast images representing the modulation of effective connectivity from the seed region. Up to this point, all procedures were conducted using the CONN toolbox. Finally, we used these contrast images and the random-effect model implemented in SPM12 to test whether any regions exhibited significant differences in effective connectivity between the LIVE and REPLAY conditions. Analyses were assessed at *p* < 0.05 with FWE correction at the cluster level. The height threshold to form each cluster was set at an uncorrected *p* value of 0.001. This relatively high cluster-forming threshold is enough to prevent the failure of a multiple-comparison problem in cluster-level statistical inference (Eklund et al., 2016; Flandin and Friston, 2017). We also listed statistical values estimated by the SnPM toolbox with a nonparametric permutation test.

**Table 2.** Regions exhibiting greater activation in the LIVE condition than in the REPLAY condition

Cluster level inference			Peak level inference		*t* value	MNI coordinates			Side	Location	Probability
P_FWE_		Cluster size mm^3^	P_FWE_								
SPM	SnPM		SPM	SnPM		*x*	*y*	*z*			
**0.015**	**0.025**	2616	0.960	0.443	3.848	−40	−60	−30	L	Cerebellum	Lobule VIIa crus I (Hem) (99%)
			0.006	0.001	6.734	−28	−46	−30	L	Cerebellum	Lobule VI (Hem) (85%)
			0.642	0.195	4.406	−28	−44	−44	L	Cerebellum	
**0.010**	**0.022**	2880	0.408	0.111	4.720	−18	−60	−52	L	Cerebellum	Lobule VIIIb (Hem) (68%)
			0.846		4.119	−6	−54	−54	L	Cerebellum	Lobule IX (Hem) (80%)
			0.954		3.870	−14	−52	−52	L	Cerebellum	Lobule IX (Hem) (67%)
			0.815	0.283	4.169	6	−56	−56	R	Cerebellum	Lobule IX (Hem) (86%)
			0.495	0.139	4.598	12	−50	−50	R	Cerebellum	Lobule IX (Hem) (87%)
**0.002**	**0.014**	4176	0.274	0.069	4.945	−8	10	50	L	Pre-SMA	
			0.986	0.532	3.702	−10	10	38	L	ACC	
			0.274	0.069	4.945	6	12	40	R	ACC	
0.056	**0.040**	1824	0.227	0.055	5.044	−8	−46	−22	L	Cerebellum	
			0.463	0.127	4.641	0	−56	−26	R	Cerebellum	Fastigial nucleus (37%)
			0.471	0.130	4.630	14	−52	−30	R	Cerebellum	

Hem, Hemisphere L, left; R, right. The *p* values satisfying the statistical threshold (*p* < 0.05) after correcting for multiple comparisons (pFWE) are emphasized using bold type.

##### Interbrain synchronization analysis

We tested for differences in the interbrain synchronization of the LIVE and REPLAY conditions using conventional voxel-to-voxel method used by previous hyperscanning fMRI studies that can identify interbrain synchronization of activation without any prior assumptions (Saito et al., 2010; Tanabe et al., 2012). We focused on the spontaneous fluctuation of BOLD signal that is unrelated to the task-related activation or deactivation (Fair et al., 2007). First, the task-related activation/deactivation was removed from the BOLD time series using the GLM model implemented in the SPM12. This yielded 3D-Nifti files representing residual time series that are independent of task-related activation/deactivation compared with baseline (i.e., the REST condition). Second, we divided the original time series into three sub-time series based on the experimental design: LIVE, REPLAY, and REST conditions. Third, we concatenated sub-time series into one long time series. The length of the LIVE- and REPLAY-related residual time series was 640 volumes. Next, we calculated the interbrain synchronization between the voxels representing the same MNI coordinates (*x*, *y*, *z*) in the two participants using the Pearson’s correlation coefficient. This computation was performed using a MATLAB script developed in-house. The correlation coefficient *r* was transformed to the standardized *z* score using Fisher’s *r*-to-*z* transformation. Finally, we obtained two 3D-Nifti images representing interbrain synchronization in the LIVE and REPLAY conditions per pair.

We conducted the random-effects model analysis in SPM12 at the group level. The normalized interbrain synchronization images were used in the group-level analysis. Here, the paired *t* test was used to test the differences in interbrain synchronization between the LIVE and REPLAY conditions. The resulting set of voxel values for each contrast constituted a statistical parametric map of the *t* statistic (SPM {t}). The threshold for significance of the SPM {t} was set at *p* < 0.05 with FWE correction at the cluster level for the entire brain (Friston et al., 1996); the height threshold was set at an uncorrected *p* value of 0.001. This cluster threshold is conservative enough to prevent failure in cluster-level inference (Eklund et al., 2016; Flandin and Friston, 2017). The statistical inference was also estimated by a nonparametric permutation test using the SnPM toolbox, like the GLM and gPPI analyses. Anatomic labeling was based on Automated Anatomic Labeling (Tzourio-Mazoyer et al., 2002) and the Anatomy toolbox version 1.8 (Eickhoff et al., 2005). Final images have been displayed on a standard template brain image (http://www.bic.mni.mcgill.ca/ServicesAtlases/Colin27) using MRIcron (https://www.nitrc.org/projects/mricron; Rorden and Brett, 2000).

## Results

### Behavioral index

Figure 3*A* shows the average number of eye-blinks per block. Repeated-measures ANOVA revealed a significant effect of condition (Table 1, a; *F*_(2,54)_ = 13.1814, *p* < 0.0001, η_g_^2^ = 0.0354). A *post hoc* comparison with Bonferroni correction revealed that there were no significant differences in the number of eye-blinks between the LIVE and REPLAY conditions (Table 1, d; *t*_(27)_ =2.3522, *p* = 0.0786, Bonferroni correction), while the number of eye-blinks was greater in the REST condition than in the LIVE (Table 1, b; *t*_(27)_ =3.9464, *p* = 0.0015, Bonferroni correction) and REPLAY (Table 1, c; *t*_(27)_ = 3.8499, *p* = 0.0021, Bonferroni correction) conditions.

**N**ext, we compared the ΣNCR values using repeated-measures ANOVA (Fig. 3*B*) and found a significant effect of condition was significant (*F*_(3,81)_ = 3.9830, *p* = 0.0295, η_g_^2^ = 0.03236; Table 1, e). A *post hoc* comparison with Bonferroni correction revealed that there were significant differences between the ${\text{ΣNCR}}_{F\to F}^{LIVE}{\text{and ΣNCR}}_{F\to F}^{REPLAY}$ (*t*_(27)_ = 3.406, *p* = 0.0126; Table 1, f), ${\text{ΣNCR}}_{F\to F}^{LIVE}{\text{and ΣNCR}}_{S\to F}^{REPLAY}$ (*t*_(27)_ =3.2934, *p* = 0.0168; Table 1, h). Differences in the other pairs did not meet the threshold for statistical significance (Table 1, g, i, j, k). To confirm that the outliers did not skew the parametric statistics, we recomputed the statistical values after removing outliers defined by two SDs rather than 1.5. Four subjects to whom the outlier data could be attributed in at least one of the four conditions were excluded from the analysis; the repeated-measures ANOVA therefore included a sample of 24. Even after removing the outliers, the repeated-measures ANOVA could replicate the significant effect of condition (*F*_(3, 69)_ = 4.3334, *p* = 0.0074, η_g_^2^ = = 0.0785; Table 1, l), as well as the significant differences between the ${\text{ΣNCR}}_{F\to F}^{LIVE}{\text{and ΣNCR}}_{F\to F}^{REPLAY}$ (*t*_(23)_ =3.0965, *p* = 0.0306; Table 1, m), and between ${\text{ΣNCR}}_{F\to F}^{LIVE}{\text{and ΣNCR}}_{S\to F}^{REPLAY}$ (*t*_(23)_ = 3.0779, *p* = 0.0318; Table 1, o). Differences in the other pairs did not meet the threshold for statistical significance (Table 1, n, p, q, r).

We also tested differences across enhanced ΣNCR values using repeated-measures ANOVA (Fig. 3*C*) and found that the effect of condition was significant (*F*_(2,54)_ = 10.3784, *p* = 0.0002, η_g_^2^ = 0.03236; Table 1, s). A *post hoc* comparison with Bonferroni correction revealed that there were significant differences between ${\text{ΣNCR}}_{F\to F}^{LIVE}{\text{-ΣNCR}}_{F\to F}^{REST}$ and ${\text{ΣNCR}}_{F\to F}^{REPLAY}{\text{and ΣNCR}}_{F\to F}^{REST}$ (*t*_(27)_ = 3.4061, *p* = 0.0063; Table 1, t), as well as between ${\text{ΣNCR}}_{F\to F}^{LIVE}{\text{-ΣNCR}}_{F\to F}^{REST}$ and ${\text{ΣNCR}}_{S\to F}^{REPLAY}{\text{and ΣNCR}}_{F\to F}^{REST}$ (*t*_(27)_ = 3.2934, *p* = 0.0084; Table 1, u). Differences in the other pair did not meet the threshold for statistical significance (Table 1, v). We recalculated statistical inferences as raw NCR values without outliers to ensure that the outliers had no effect on the inferences. The stricter criteria for outliers remained 2 SDs, resulting in the removal of seven subjects from the analysis. Even after outliers were excluded from the analysis, we obtained qualitatively identical results: significant effect of condition (*F*_(2,40)_ = 7.9233, *p* = 0.0013, η_g_^2^ = 0.1330; Table 1, w), and significant differences between ${\text{ΣNCR}}_{F\to F}^{LIVE}{\text{-ΣNCR}}_{F\to F}^{REST}$ and ${\text{ ΣNCR}}_{F\to F}^{REPLAY}{\text{-ΣNCR}}_{F\to F}^{REST}$ (*t*_(20)_ = 2.8343, *p* = 0.0306; Table 1, x) and between ${\text{ΣNCR}}_{F\to F}^{LIVE}{\text{-ΣNCR}}_{F\to F}^{REST}$ and ${\text{ΣNCR}}_{S\to F}^{REPLAY}{\text{-ΣNCR}}_{F\to F}^{REST}$ (*t*_(20)_ = 2.9034, *p* = 0.0265; Table 1, y). Differences in other pairs did not meet the threshold for statistical significance (Table 1, z).

To test whether or not these enhancements of entrainment of eye-blinking is influenced by the number of blocks, we calculated the Akaike causality index for separate blocks of the experiment and applied the repeated-measures ANOVA (4 blocks × 4 conditions) to the ΣNCR data. We found a significant effect of conditions (*F*_(3,81)_=3.9830, *p* = 0.0106, η_g_^2^ = 0.0132; Table 1, aa). However, the effects of sessions (*F*_(3,81)_=1.0351, *p* = 0.3816, η_g_^2^ = 0.0139; Table 1, bb) and interaction (session × conditions; *F*_(9,243)_ = 1.8235, *p* = 0.0647, η_g_^2^ = 0.0128; Table 1, cc) were nonsignificant. Therefore, in the following analysis of neuroimaging data, we combined data from the four blocks.

### Brain activation in the LIVE and REPLAY conditions

**Figure 4.**
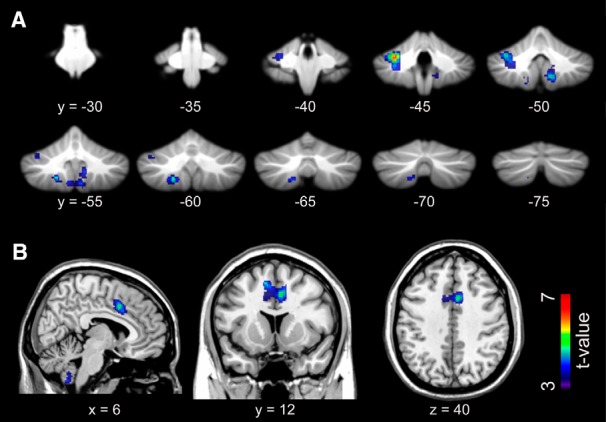
Brain regions exhibiting significantly greater activation in the LIVE condition than in the REPLAY condition. ***A***, Cerebellar activation is overlaid on the coronal planes of the SUIT template (Diedrichsen, 2006; Diedrichsen et al., 2009). ***B***, The activation in the ACC is superimposed on the T1-weighted high-resolution anatomic MRI normalized to the MNI template space in the sagittal (left), coronal (middle), and transaxial (right) planes that crossed at (6, 12, 40) in the MNI coordinate system (in mm). SUIT, Spatially unbiased infratentorial template.

We used GLM analysis (Table 1, dd, ee) to elucidate brain activation in the LIVE and REPLAY conditions. For the LIVE versus REPLAY contrast, we observed greater activation in the left cerebellar hemisphere (lobules VI, VII, and VIIIa), bilateral paravermis area (lobule XI; Fig. 4*A*), and the pre-supplementary motor area (SMA) extending to the dorsal tier of the anterior cingulate cortex (ACC; Fig. 4*B*). No significant differences in activation were observed in the REPLAY versus LIVE contrast. Detailed information regarding each cluster is outlined in Table 2.

### Results of the gPPI analysis

**Figure 5.**
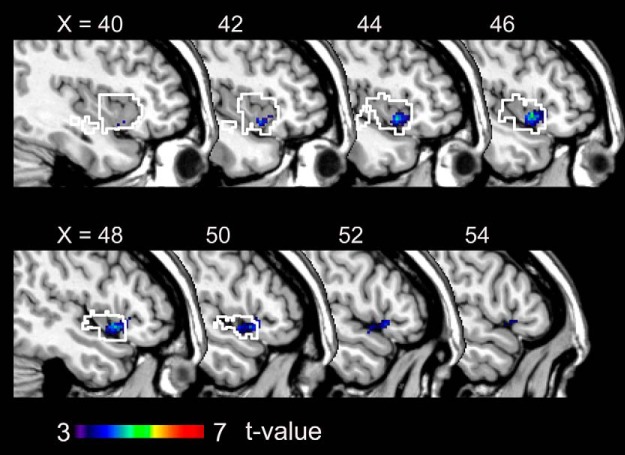
Regions exhibiting greater effective connectivity from the ACC in the LIVE condition than in the REPLAY condition. The area outlined in white is the dAIC (Chang et al., 2013). X indicates the MNI coordinates (in mm).

The gPPI analysis (Table 1, ff, gg) revealed that the effective connectivity from the ACC region toward the dorsal anterior insular cortex (dAIC; Chang et al., 2013) was greater during the LIVE condition than during the REPLAY condition (Fig. 5, Table 3). No regions exhibited greater effective connectivity involving the pre-SMA-ACC regions in the REPLAY condition than in the LIVE condition. There was no modulation of effective connectivity involving cerebellar seed regions.

**Table 3.** Regions exhibiting enhanced effective connectivity from the ACC in the LIVE condition

Cluster level inference			Peak level inference		*t* value	MNI coordinates			Side	Location	Probability
P_FWE_		Cluster size (mm^3^)	P_FWE_								
SPM	SnPM		SPM	SnPM		*x*	*y*	*z*			
**0.000**	0.0824	1208	0.868	0.378	5.063	46	14	−6	R	Insula	
			1.000	1.000	3.545	54	14	−4	R	IFG	BA44 (21%)
			1.000		4.156	50	20	−4	R	IFGOr	BA45 (31%)

IFG, Inferior frontal gyrus; IFGOr, Inferior frontal gyrus (pars opercularis); BA, Brodmann area; R, right. The *p* values satisfying the statistical threshold (*p* < 0.05) after correcting for multiple comparisons (pFWE) are emphasized using bold type.

### Interbrain synchronization

**Figure 6.**
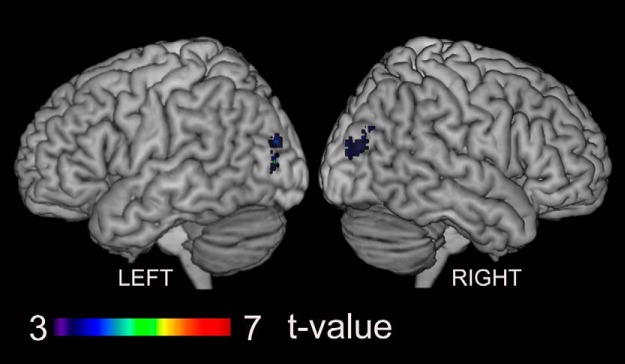
Regions exhibiting greater interbrain synchronization during the LIVE condition than the REPLAY condition. These areas are superimposed on a surface-rendered high-resolution anatomic MRI normalized to the MNI template viewed from the left and right.

Figure 6 illustrates interbrain synchronization that is specific to the LIVE condition (Table 1, hh, ii). It was found on the bilateral middle occipital gyrus (MOG). Detailed information about these clusters is described in Table 4. No regions showed significant interbrain synchronization in the REPLAY condition compared with the LIVE condition.

**Table 4.** The regions exhibiting enhanced interbrain synchronization in the LIVE condition compared with REPLAY condition

Cluster level inference			Peak level inference		*t* value	MNI coordinates			Side	Location	Probability
P_FWE_		Cluster size (mm^3^)	P_FWE_								
SPM	SnPM		SPM	SnPM		*x*	*y*	*z*			
**0.001**	0.2258	1088	0.999	0.829	5.753	−26	−82	4	L	MOG	
			1.000	0.999	4.695	−34	−78	4	L	MOG	
			1.000	0.999	4.628	−28	−86	22	L	MOG	
**0.007**	0.2852	880	1.000	0.998	4.739	28	−76	24	R	MOG	
			1.000	1.000	3.983	38	−80	16	R	MOG	
			1.000	1.000	3.827	34	−88	18	R	MOG	hOc4lp (35.4%)

L, Left; R, right. The *p* values satisfying the statistical threshold (*p* < 0.05) after correcting for multiple comparisons (pFWE) are emphasized using bold type.

## Discussion

This study aimed to elucidate the behavioral and neural representations of mutual interaction during eye contact by comparing the neural activity associated with real-time eye contact with that associated with non-real-time eye contact. Our findings suggest that mutual interaction/shared attention during eye contact is mediated by the cerebellum and the limbic mirror system.

### Behavioral index

In this study, causal analysis using an MVAR model (Akaike, 1968; Ozaki, 2012) was performed to assess how an individual’s temporal attentional window is influenced by that of the partner (Schippers et al., 2010; Okazaki et al., 2015; Leong et al., 2017). Our results show that participants were more sensitive to the eye-blinks of a partner in the LIVE condition than in the REPLAY condition because none of the participants perceived the difference between the LIVE and REPLAY conditions. Thus, the experimental setup for our LIVE condition enabled a reciprocal feedback system through the visual modality. Our findings suggest that perceptual–motor interaction occurs during eye contact without conscious awareness. Previous researchers have argued that an essential component of real-time social interactions involves reciprocal coupling via perceptual–motor linkages between interacting individuals (Nicolis and Prigogine, 1977; Haken, 1983; Bernieri and Rosenthal, 1991; Strogatz, 2003; Oullier et al., 2008). Our results extend this notion to the attention mediated by the minimal motion of blinking, which represents the temporal window of attention toward one’s partner. Interestingly, the influence from a partner was significantly greater when the information flow between two individuals was reciprocal (${\text{ΣNCR}}_{F\to F}^{LIVE}$) than when it was unidirectional (${\text{ΣNCR}}_{S\to F}^{REPLAY}$). As the mutual interaction in real time evinced a significant effect on the partner’s eye-blink, this finding indicated that the mutual on-line interaction is critical to the influence of the other’s eye-blink. Feedback through the on-line mutual interaction may induce a nonlinear response, causing the subtle effect to be amplified (Okazaki et al., 2015).

This experiment can be regarded as a simplified version of the social contingency detection task originally reported by Murray and Trevarthen (1985). Social contingency is defined as the cause–effect relationship between one’s behavior and consequent social events (Gergely, 2001; Nadel, 2002) and is highly associated with a sense of self or one’s own body in infancy, developing a sense of reciprocity, and participation with others (Rochat, 2001), all of which are critical for typical development (Mundy and Sigman, 1989; Gergely, 2001; Goldstein et al., 2003; Kuhl et al., 2003; Watanabe, 2013). Several previous studies have investigated differences in mother–infant interactions between real-time bidirectional interaction and off-line unidirectional interaction (Murray and Trevarthen, 1985; Nadel, 2002; Stormark and Braarud, 2004; Soussignan et al., 2006). Even in adults, turn-taking behavior accompanying social contingency is likely to serve as experience sharing, which represents the basis of all social behaviors (Rochat et al., 2009; Stevanovic and Peräkylä, 2015). Our results indicate that even a minimal task condition, such as mutual gaze, constitutes a reciprocal feedback system that can provide a basis for the detection of social contingency, promoting sharing of attention between partners (Farroni et al., 2002; Schilbach, 2015).

### Neural substrates of eye contact in real time

Using a conventional GLM approach, we observed LIVE-specific activation in the cerebellum and ACC. The cerebellum plays a key role in error detection and processing of temporal contingency (Blakemore et al., 2003; Trillenberg et al., 2004; Matsuzawa et al., 2005), the latter of which is critical for real-time social communication (Gergely and Watson, 1999). The cerebellum is also critically involved in sensorimotor prediction (Blakemore and Sirigu, 2003), especially in building predictions about the actual sensory consequences of an executed motor command. One previous fMRI study reported that the prediction error caused by sensory feedback is essential for acquiring internal forward models of movement control (Imamizu et al., 2000). This prediction (forward model) is mainly used in the early stages of movement execution to maintain accurate performance in the presence of sensory feedback delays (Wolpert and Kawato, 1998), as well as in social interaction (Wolpert et al., 2003). Considering that real-time social interaction can be regarded as a cross-individual sensorimotor loop (Wolpert et al., 2003; Froese and Fuchs, 2012), the cerebellum may receive visual afferents of the partner’s blink as sensory feedback for the prediction of one’s blink movement, to evaluate temporal contingency between the partners’ blinks.

In humans, the ACC is located in the medial wall of the cerebral hemisphere, adjacent to the pre-SMA (Habas, 2010). The ventral (limbic) tier occupies the surface of the cingulate gyrus, corresponding to Brodmann’s areas 24a and 24b, and subcallosal area 25. The dorsal (paralimbic) tier is buried in the cingulate sulcus, corresponding to Brodmann’s areas 24c and 32 (for review, see Paus, 2001). The dorsal tier is involved in volitional motor control (Deiber et al., 1996; Picard and Strick, 1996; Brázdil et al., 2006).

The ACC and cerebellum constitute a tightly connected corticocerebellar network. Recent functional connectivity analysis studies have demonstrated that distinct cerebellar seed regions in the anterior portion of the crus I exhibit functional connectivity with the dorsolateral prefrontal cortex, the rostral portion of the inferior parietal lobule, and a frontal midline region bordering the pre-SMA and ACC in healthy adults (Buckner et al., 2011; Riedel et al., 2015). Conversely, the ACC exhibits a negative correlation with the cerebellum (Margulies et al., 2007), possibly reflecting its hypothesized role in the inhibition of prepotent stereotyped responses (Paus et al., 1993; Paus, 2001). In terms of anatomic connectivity, Zalesky et al. (2014) used diffusion MRI to demonstrate disruption of WM connectivity between the cerebellum and the cingulate cortex in individuals with Friedreich ataxia, an autosomal recessive disease involving degeneration of the spinal cord and cerebellum, thereby supporting the notion of reverse cerebellar diaschisis (Schmahmann and Sherman, 1998).

The corticocerebellar–thalamocortical circuit involving the cerebellum and ACC plays a role in attention. The cerebellum is involved in attention, including anticipation/prediction of the internal conditions for a particular operation, as well as the setting of specific conditions in preparation for that operation (Allen et al., 1997; Schweizer et al., 2007). Honey et al. (2005) reported that patients with schizophrenia exhibited an attenuated response of the ACC and cerebellum to degradation of the target during a continuous performance task, paralleling their limited visual attentional resources. They also observed disruption in the pattern of task-related connectivity of the ACC to the prefrontal regions. Honey et al. (2005) concluded that attentional impairments associated with schizophrenia could be attributed to the corticocerebellar–thalamocortical circuit, which includes the ACC and cerebellum. Considering the role of the ACC and cerebellum in sensorimotor and attentional control, the ACC–cerebellar network may constitute a reactive–predictive controller system (Noy et al., 2011) by which one’s own attention-contingent motor output (that is, eye-blink) is modulated by the visual input of the partner’s movement. Under the mirror configuration during the LIVE condition, the reactive–predictive controllers in two individuals work to coordinate their own behavior with the partner’s. Thus, it closes the sensorimotor circuits across the individuals.

### Enhanced connectivity between the ACC and AIC

We observed enhanced effective connectivity from the ACC to the right dAIC in the LIVE condition than in the REPLAY condition. In the present study, no emotional processes were included in the task, suggesting that the enhancements in connectivity were related to recurrent interaction via eye contact. The ACC has a strong connection to the AIC (Margulies et al., 2007; Ghaziri et al., 2015), most prominently in the dAIC (Chang et al., 2013), a central hub in which several different cognitive networks converge (Dosenbach et al., 2006; Chang et al., 2013). The ACC–AIC network represents the portion of the limbic mirror system related to the recognition of affective behavior (Singer et al., 2004; Fabbri-Destro and Rizzolatti, 2008; Cattaneo and Rizzolatti, 2009).

Medford and Critchley (2010) proposed that the AIC and ACC represent the basis of self-awareness by constituting the input (AIC) and output (ACC) components of a system. In such a system, the integrated awareness of cognitive, affective, and physical states first generated by the integrative functions of the AIC are then re-represented in the ACC as a basis for the selection of and preparation for responses to inner or outer events. Craig (2009) regarded the AIC as the probable site for awareness, based on its afferent representation of “feelings” from the body, and the ACC as the probable site for the initiation of behaviors. Meltzoff (2005) proposed a “like-me” framework for the understanding of others. He suggested that imitation enables the understanding of another mind based on an understanding of actions and their underlying mental states. Singer et al. (2004) observed that pain empathy relies on neural structures that are also involved in the direct experience of that emotion [i.e., the limbic mirror system (ACC, AIC)]. This finding is consistent with the Simulation Theory, which proposes that “we understand other people’s minds by using our mental states to simulate how we might feel or what we might think in a given situation” (Lamm and Singer, 2010). Lamm and Singer (2010) concluded that perceiving the states of another activates neural representations encoding each state when it is experienced personally. In the eye-contact state, participants are aware that they are attending to their partner during eye contact. Therefore, given that the ACC–AIC network represents self-awareness, its activation during real-time eye contact may represent a shared mental state (i.e., awareness involving the participant and partner) such as shared attention. This interpretation is consistent with a study by Hietanen et al. (2008), which demonstrated that autonomic arousal is enhanced by eye contact with a live human, but not with static images of faces. The authors argued that this might be due to the enhancement of self-awareness by the presence of another person. The results of our study suggest that the self-awareness is enhanced by the social contingency generated with live humans through the interaction of each other’s attentional windows via eye-blinks and that the regulation of self-awareness by interaction might be caused by the cerebellar–cerebral networks that tap into the limbic mirror system.

### Interbrain synchronization

By comparing the degree of interbrain synchronization between the LIVE and REPLAY conditions, we found an enhancement in the MOG region related to the LIVE condition. This region is in the lateral occipitotemproral cortex (LOTC) and is almost identical to the region that shows interbrain synchronization specific to the eye-contact state (Koike et al., 2016). Previous studies suggest that the LOTC receives both sensory inputs of a partner’s behavior (Lingnau and Downing, 2015) and efference copies of one’s own behavior (Astafiev et al., 2004; Orlov et al., 2010). Therefore, the roles of the LOTC in supporting action perception and overt action performance are closely related. The LOTC may play a role in the human action observation network (Caspers et al., 2010) that is typically attributed to the frontoparietal mirror system (Oosterhof et al., 2013). Thus, the MOG region may conceivably receive information about self and other’s eye-blinks.

Based on the electroencephalography (EEG) hyperscanning experiment of the mutual gaze between mothers and infants, Leong et al. (2017) found interpersonal neural synchronization. They argued that the phase of cortical oscillations reflects the excitability of underlying neuronal populations to incoming sensory stimulation (Schroeder and Lakatos, 2009), a possible mechanism for temporal sampling of the environment (Giraud and Poeppel, 2012). Interpersonal neural synchronization could increase within a dyad during the course of social interaction because each partner is continuously producing salient social signals (e.g., gaze) that act as synchronization triggers to reset the phase of his or her partner’s ongoing oscillations (Leong et al., 2017). The present study showed neural synchronization in the LOTC, which receives both visual input of others’ actions and efference copies of one’s own actions. The salient social signals were sent to the partner through gaze or blink (defining the temporal attentional window), and the motor command corresponding to which is likely delivered to the LOTC as an efference copy. The eye-blink may, thus, act as a synchronization trigger. Therefore, the cross-individual neural synchronization of the MOG represents the alignment of the temporal pattern of attention, which may optimize communicative efficiency (Leong et al., 2017).

### Limitations and future directions

The present study is subject to several limitations. First, concerning the hyperscanning fMRI experimental design, the very long mutual gaze condition was not ecological and may be quite different from conceptions of “mutual gaze” or “eye contact” informed by daily life. This is due to our use of a blocked design, the most effective way to detect brain activation. Also, the product of our experimental design, estimations of the temporal dynamics of eye-blink entrainment, brain activation, and interbrain synchronization, could not be performed. While we could not find a significant effect of session on the eye-blink entrainment in real-time eye contact, it is possible that the eye-blinking entrainments only occur in the very first phase of mutual gaze condition in one block. By refining the experimental and analytical design, we may further gain insight into the dynamics of interindividual interaction through eye-contact and interbrain synchronization. To explore the temporal dynamics of interbrain synchronization, we are currently conducting a hyperscanning simultaneous EEG-fMRI recording that could integrate the merits of the two neuroimaging methods (Koike et al., 2015). As the present study demonstrated the efficacy of using Akaike causality analysis to evaluate dynamic mutual interaction, future studies applying this method to EEG data in ecological settings of normal and diseased populations are warranted.

The present study is also limited by its capacity to find interbrain synchronization only between homologous regions, but not between nonhomologous regions (i.e., frontoparietal synchronization; Dumas et al., 2010). In our setting, two participants play identical roles in eye-to-eye communication; therefore, the resonance through interbrain closed loop might occur in the homologous regions. However, the interbrain effect may also occur between nonhomologous regions. To explore this possibility, an ROI analysis based on the precise parcellation of human cerebral cortex in a human connectome project may be the most suitable (Glasser et al., 2016). Future studies adapting this method could reveal the mechanism underlying the means by which two brains are wired through eye-to-eye communication without any conscious awareness.

### Summary

In the present hyperscanning fMRI study, we focused on real-time mutual interaction during eye contact. The open-and-close timing of the attentional window, defined by eye-blinks, was entrained to that of the counterpart during real-time mutual interaction. Our findings indicate that the social interaction is nonlinear, and the influence from the partner might be amplified by the nonlinearity during the real-time interaction. Corresponding with the nonlinearly amplified behavioral coordination, real-time interaction during eye contact was found to be mediated by the amplified activation of the cerebellum and the cingulate motor cortex. This was accompanied by enhanced connectivity within the limbic mirror system. These findings underscore the notion that real-time eye contact generates an emergent property of shared attention, which is mediated by a cerebellocerebral network inclusive of the limbic mirror system.

## Synthesis

Reviewing Editor: Satu Palva, University of Helsinki Decisions are customarily a result of the Reviewing Editor and the peer reviewers coming together and discussing their recommendations until a consensus is reached. When revisions are invited, a fact-based synthesis statement explaining their decision and outlining what is needed to prepare a revision will be listed below. The following reviewer(s) agreed to reveal their identity: Edda Bilek.

Synthesis

In this manuscript the authors used live hyperscanning to specifically test for social interaction between two participants. This is a very important study as it has been argued that only through naturalistic two-persons set-up we can properly elicit those mirroring areas involved in social interaction. This manuscript summarizes the findings of a passive viewing study, where subjects engaged in eye contact over an extended amount of time. The study included two control conditions: delayed video presentation and a no viewing condition. One main read-out is the noise contribution ratio, quantifying how much the eye-blink time-series of one subject influenced the other, and a significant association was found for immediate contact only. In addition, increased connectivity between ACC and anterior Insula was reported for the live contact condition.

The major strengths of this paper are the high standard of technical equipment the authors used, the choice of control data, as well as the implementation of metrics allowing for the inference of causal relationships across subjects. The study has been carefully designed and the methodological approaches are justified. The level of transparency of the obtained results should, however, be increased to convince the readers. Specifically the data should be presented in more comprehensive way.

Major

- The authors conduct a costly hyperscanning fMRI study but use hyperscanning only to examine eye-movement data but not the fMRI data in a two-brain manner. There is now a good amount of studies showing that hyperscanning data allows for the computation of some form of multi-brain coherence (for examples King-Casas, 2005; Cui, 2012; Bilek et al., 2015). The lack of these analyses is a clear weakness in the study and should be applied to the neuroimaging data as well as. These data should be reported even in the case of non-significant results.

- it has been shown that bar plots used in Figure 3 are misleading (see https://journals.plos.org/plosbiology/article?id=10.1371/journal.pbio.1002128).

Hence, it would be crucial to replace Figure 3 with a swarm plot OR box plot OR violin plot. A recent way to visualize the data includes all of these plots into one (raincloud plots https://micahallen.org/2018/03/15/introducing-raincloud-plots/ ). As you are matlab users, also using the gramm toolbox for these kind of plots that showing the full range of values in your data is recommended. https://github.com/piermorel/gramm

The results (or Figure 3) are also missing a comparison of absolute NCR values (i.e., condition specific as shown in Figure 3A for the NCR values of Figure 3B).

- SPM in a GLM with widely used choices were used for statistical inference. Yet, these methods do not true fully correct for the multiple comparisons performed. Permutation based methods are more robust and produce results closer to the actual false discovery rate assumed in the data (http://www.pnas.org/content/113/28/7900) and should hence be used to obtain corrected statistical level. One option would be to re-run the SPM analysis using the SnPM toolbox https://warwick.ac.uk/fac/sci/statistics/staff/academic-research/nichols/software/snpm for non-parametric permutation based analysis. This means that only the group level analysis should be re-run for the case shown in figure 4. It might be that the permutation based approach will lead to no significant clusters. The authors can still report their current results, however they should also add the results obtained with SnPM for a more transparent reporting.

- The \SigmaNCR results have been sometimes found to be a bit weak. Could there be a dynamic component that the average is not going to grasp. I.e. the leader and follower in the gazing might dynamically change in a sort of a dialogue/interaction. Do you think that averaging of NCR loses important time-varying information? A short note about this could be added in the discussion - The NCR would be a good covariate in the analysis of fMRI data. Especially since the authors conclude that “the enhancements in connectivity were related to recurrent interaction via eye contact”.

- The choice of ROIs (ACC and anterior Insula) implies a focus on the attention system rather than a social network. For a closer look on social functioning, right TPJ/pSTS and mPFC would be interesting as additional ROIs, as they are commonly found as regions involved in mutual gaze tasks and interaction. The selection of regions is quite important here, since the authors conduct the gPPI analysis for these regions only. Therefore, the manuscript would benefit from an examination of these regions or the PPI could be reported on a whole brain level before the ROI analyses.

- The term “interaction” is used in a misleading way. Interaction implies some form of exchange between subjects. Eye-blinks (not to be mistaken with general mimic or communicative eye-movements) are usually unconscious motor movements that serve non-social biological causes. I understand, however, that the frequency is altered by attention, and attention is altered by social cues. Therefore, there may be an association between the number of eye-blinks and social cues. Please give an introduction into why the synchronization of eye blinks is necessary for human social interaction. To the reader, this may seem like a random measure given the large amount of data the authors acquired. Related, please explain why blinks represent an attentional window. Does the attention stop during the 150ms of the blink and is reoriented somewhere else after? Doesn't the task allow only for attention to the partner?

- The tasks involved eye-contact for an extended amount of time (4*8.3/3 minutes). What exactly did subjects do during this time? Continuously staring at a conspecific is a very unusual and sometimes quite uncomfortable scenario. In certain species, this might even suggest aggression towards the opponent. Subjects likely either included some form of communication (via mimicry, e.g., making faces) or interrupted the eye-contact. Also, what was the task instruction to the subjects?

- Did the number of eye-blinks change over time? For a measure of interaction, I would expect the synchronization to increase to a plateau. For a measure of arousal or alertness, I would expect a decline.

- If two subjects synchronize their eye-blinking, why did one subject not synchronize their blinking with the video?

- How was the linear trend modeled exactly (briefly mentioned in the first section of behavioral data analysis)

- The paragraph starting with line 214 is a little confusing. Brain function is mentioned, although this is the behavior section. Then, a result is referenced (i.e., a lack of synchronization) for unknown reasons. This may be rephrased or removed.

- The discussion is a bit long and could be slightly shortened and more focused on the core findings.

- The authors are encouraged to upload the unthresholded 3D NIFTI maps used to produce Figure 4 and Figure 5 to NeuroVault.org. This is important not only for future meta-analysis, but also because it will increase the popularity of the paper after its publication.
